# Simulation of the trimeric globular head of C1q reveals temperature-sensitive network: implications for inflammation

**DOI:** 10.1007/s00894-025-06464-y

**Published:** 2025-08-13

**Authors:** Nicole Rodgers, Christophe Lalaurie, Thomas Christopher Richard McDonnell

**Affiliations:** 1 Division of Medicine, University College London, London, WC1E 6JF UK; 2 The Department of Aging, Rheumatology and Regenerative Medicine, Division of Medicine, University College London, London, WC1E 6JF UK

**Keywords:** Simulation, C1q, Inflammation, Complement, Flexibility, Trimer

## Abstract

**Context:**

C1q is an important protein in immune processes, driving complement activation through the classical pathway. Further to this, alterations in C1q either through SNPs or through autoantibodies can lead to systemic lupus erythematosus. Beyond these functions, C1q can also bind to other inflammatory proteins such as C-reactive protein (CRP) via its globular domain, when CRP is in the pentameric form. These interactions require specific structures to facilitate binding. Using molecular dynamics simulations, it is possible to measure the movements of proteins over time, with increasing temperatures allowing them to explore most of their available conformational space. Here, we describe using an increasing temperature simulation of C1q to identify potential structures generated during states of increased energy such as inflammation. Increasing temperature yielded significantly more movement of the monomeric and trimeric protein forms. Monomer A drove most movement within the molecule regardless of temperature, within the monomer and trimer. Further to this, novel structures were generated at higher temperatures, with significant movement of the CRP binding site. The altered movement in the CRP binding amino acids was correlative with increased temperature driving a loss of correlation between the different amino acids involved. Increased temperature and energy in the system leads to an alteration of C1q’s structure, which may leave it unable to bind to CRP in solution. This could have implications for the activity of the C1q/CRP complex as well as both proteins individually.

**Methods:**

Models were generated using PDB:1PK6 and prepared using Charmm-GUI’s online platform. Protein simulations were run using NAMD on the UCL HPC facility (ARC). Trajectories were combined and aligned for analysis and visualised using Visual Molecular Dynamics (VMD). Analysis was carried out using VMD, R Studio, and Excel to identify novel structures of C1q, areas of increased flexibility, and potential protein networks.

## Introduction

**Fig. 1:**
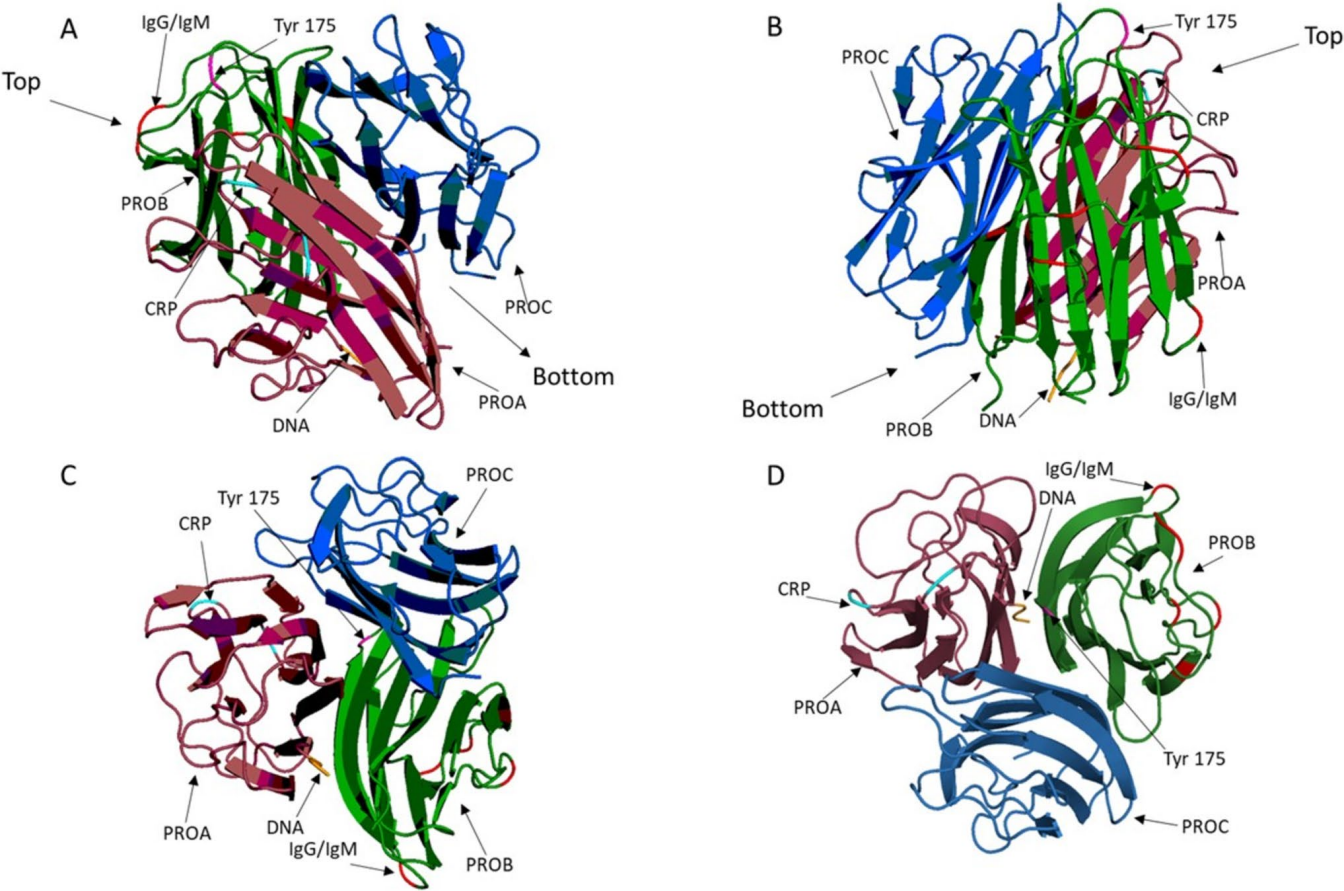
C1q trimer visualised in PyMOL with the monomers individually coloured. PROA, dark red; PROB, green; PROC, blue. Known binding sites from literature are shown; IgG and IgM, red; C-reactive protein, cyan; Tyr 175 (found in both CRP and IgM sites), pink; and secondary DNA, orange (the remaining secondary binding site and primary binding site are located on the non-visualised collagen-like region of C1q). Different 2D orientations of the trimer are visualised; Panel **A** represents the globular head at a 45-degree angle, ‘Bottom’ is where the rest of the C1q protein binds. Panel **B** represents panel **A** rotated to the right 180°. Panel **C** represents the plane view of the globular head. Panel **D** views the globular head from below (panel **C** flipped 180°)

Complement Protein 1 Subunit Q (C1q) is the first subunit in the complement cascade, forming a complex with C1s and C1r to allow antibody-driven complement activation. It is approximately 410 kDa in size and features a globular head and an extended collagen-like region. The molecule exists and functions as a trimer in solution and is capable of binding a number of molecules including C-reactive protein (CRP), DNA, immunoglobulin G (IgG), and immunoglobulin M (IgM) (Fig. 1). This pattern of interaction shows it has the potential to have other functions beyond generating complement activation.

The range of potential functions for C1q is best shown when it is disrupted, most notably in the disease systemic lupus erythematosus (SLE). In SLE, both antibodies to C1q and a genetic lack of C1q are seen and correlate with specific clinical outcomes such as lupus nephritis [1, 2]. Low C1q levels and anti-C1q antibodies are associated with general flare, whilst anti-C1q antibodies are theorised to occur when overload of apoptotic debris is failed to be cleared leading to aberrant antibody production [3, 4]. Antibodies to C1q traditionally target the collagen-like region of the molecule, altering complement activation; however, some research has been carried out identifying regions of the globular head which may be antigenic highlighting specific monomeric regions of interest [5].

The development of autoantibodies to C1q suggests the potential for novel structures, which may reveal cryptic neo-epitopes. Some studies [6] identified a range of sites which dynamically changed across the course of disease, with a number of cryptic neo-epitopes identified. The changes in structure to expose these epitopes were hypothesised to take place during immobilisation to a surface; however, inflammation itself has been shown to increase the energy in a local system and therefore has the potential to alter protein structure too [6].

Another interesting function of C1q is to bind the inflammatory marker C-reactive protein. CRP is an acute inflammatory protein with two structures, a pentameric ring and a monomeric subunit. The interaction of CRP with C1q can generate classical complement activation directly [7] dependent on its structure [8], whilst McGrath et al. showed in 2006 that C1q interacts using its globular head region primarily [9]. The C1q/CRP complex has independent functions, for which Sjowall [10] and Biro [11] showed that the interaction occurs in the fluid phase. This interaction can regulate complement activity in a dose-dependent manner in the monomeric form. Given the role CRP and C1q play in different autoimmune and inflammatory scenarios, the direct co-regulation of function is extremely interesting.

That said, it is interesting to note that whilst high CRP is present in a number of autoinflammatory diseases including rheumatoid arthritis [12, 13], high CRP levels are not commonly associated with SLE. Interestingly, it has recently been suggested that the role of CRP in SLE may be more complex and subtle than previously thought [14] including the potential for it to be an autoantigen [15, 16]. There is also a negative association between interferon gene expression, which has been shown to drive SLE, and the expression of CRP [17, 18].

Studying structural dynamics is extremely difficult, especially for complex molecules such as C1q where monomeric and trimeric dynamics may not be immediately noticeable. To overcome this, we can use molecular dynamics simulations to identify novel structures in proteins when exposed to stimulus [19–21]. It is common place to run simulations at a range of temperatures, because increasing the energy in a system allows proteins to adopt rare structures more frequently, thus facilitating a more complete exploration of its conformational space [22, 23]. Increasing temperature can also mimic the effects of inflammation where increased energy is exerted into a system.

We therefore aimed to carry out a comprehensive simulation of C1q’s globular head in solution at a range of temperatures to identify rare structures accessible only in higher energy states which may give an insight into the role of C1q in SLE during flares.

### Methods

#### Generation of structures

The Glycan Reader and Modeler at the CHARMM-GUI website (http://www.charmmgui.org/) [24–26] was used for molecular dynamics of the crystal structure coordinates of the globular head of C1q (PDB ID: 1PK6) [27].

#### Simulation

A theoretical water box at least 10 Å from the protein in each axis was assembled using the.

CHARMM TIP3 model for explicit water molecules, and 0.15 M NaCl was added. All calculations were performed at 303.15 k, 323.15 k, and 343.15 k in separate simulations (*n* = 10) with the use of the CHARMM36 force field. All simulations used a time step of 2 femtoseconds (fs). Equilibration used the CHARMM36 forcefield to relax particle number, volume, and temperature (NVT) conditions. Electrostatic forces were calculated using the Particle Mesh Ewald algorithm, with a force-switching function, switched off van der Waal interactions at 10 Å. Positional restraints for backbone and side chain heavy atoms were applied to ensure gradual equilibration of the system, and there was a gradual reduction in restraint forces during equilibration. Simulations were carried out using NAMD to further simulate each system for 140 ns (303 k), 150 ns (323 k), and 150 ns (343 k), using the CHARMM36 force field. The Langevin coupling coefficient was set to 1 picosecond (1 ps) for the production NPT simulation, and a constant pressure (1 bar) was maintained using a Nosé-Hoover Langevin piston with a 50 fs application and a 25 fs decay. Trajectory coordinates were saved every 100 ps, keeping the calculation timestep at 2 fs, and electrostatic interactions were updated every 20 fs. A 12 Å cut-off was used when short-range non-bonded and electrostatic interactions were calculated.

#### Analysis

Trajectories were prepared, aligned, and initially analysed using the Visual Molecular Dynamics (VMD) programme. The VMD TK Console function was utilised to compress outlier frames. R Studio was used to run scripts using R to analyse RMSD, root mean square fluctuation (RMSF), radius of gyration (*R*_*G*_), principle component analysis (PCA), and dynamic cross correlation matrix (DCCM). Plots and CSVs were outputted for further analysis. DCCM CSV data for both trimer and monomers at each temperature were graphed in Excel to identify residues of large correlative difference across temperature. Further CSV analysis in PRISM generated RMSD, RMSF, *R*_*G*_ graphs and respective histograms, and frames, RMSD, and R_G_ by cluster graphs. PyMOL was used to visualise the globular head of the C1q trimer and enabled highlighting of known binding site regions. PDB files of each PCA midpoint were obtained using VMD and then layered within PyMOL, aligned by PROA residues 92–120. PDB files for each monomer were submitted to Discotope to identify likely epitope regions. PyMOL was used to visualise the Discotope hits in cartoon, mesh, and surface form. CSVs from Discotope were used to create individual monomer and altogether Discotope graphs in PRISM.

## Results

### Higher temperatures facilitate greater movement

**Fig. 2:**
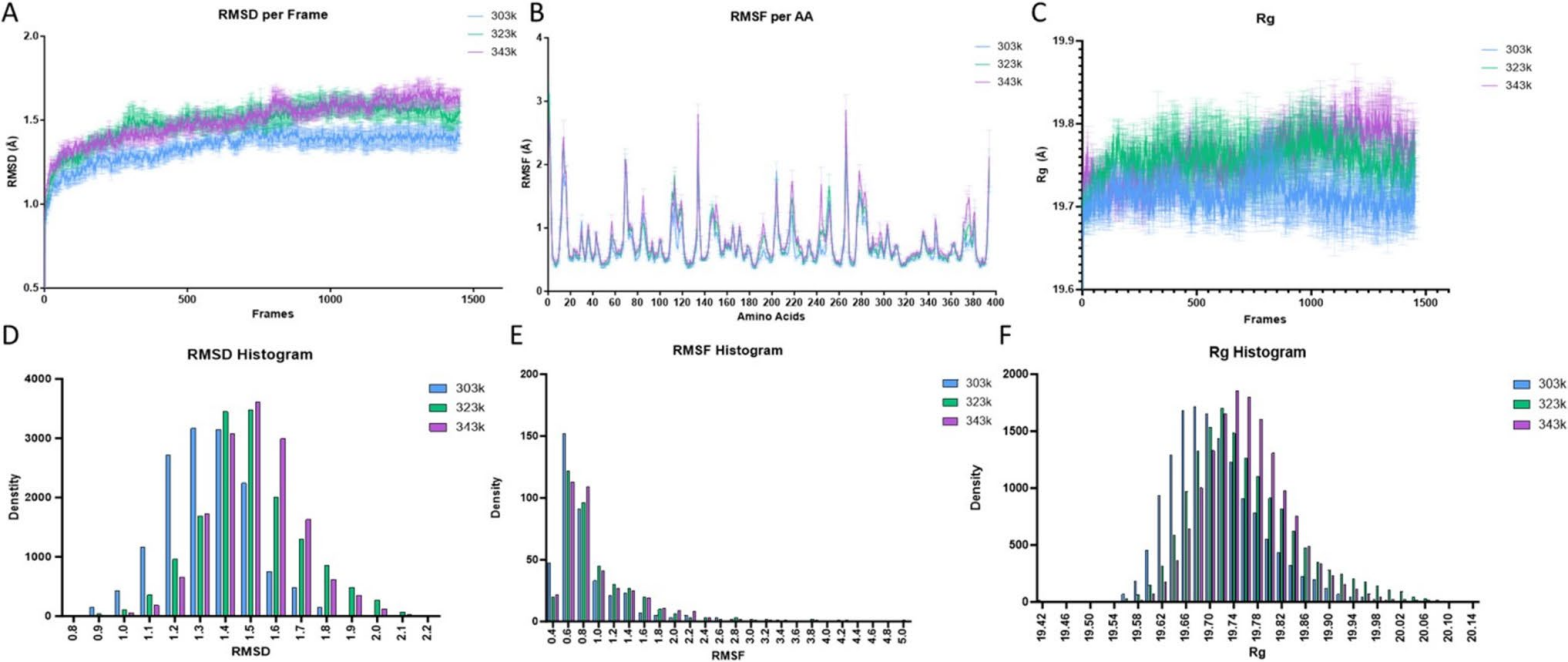
Panel **A** demonstrates global movement from starting structure for each temperature, higher temperatures plateau earlier (323 k green, 343 k purple) suggesting these simulations have more movement, thus sampling a greater conformational space. Panel **B** contains global RMSF measurements with no major differences seen between temperatures, suggesting no specific amino acid is driving structural shift. Radius of gyration (panel **C**) highlights minimal difference in overall globular shape/size with a slight, non-significant trend for higher temperatures to show larger sizes. Interleaved histograms of RMSD (panel **D**) demonstrate a differential distribution of high RMSD frames correlating with increased temperature, whilst this is not seen in panel **E** (RMSF histogram). Panel **F** contains an Rg histogram showing the minimal differential distribution of size associated with temperature

Simulations were aligned to their respective 1 st frame and movement assessed using backbone RMSD. Although overall RMSD was low (< 2 A), the higher temperature simulations (323 k, 343 k) exceeded the RMSD for 303 k within the 1 st 10 ns and remained higher throughout the simulation (150 ns) across all repeats (Fig. 2A), suggesting increased RMSD was associated with increased temperature. A histogram of RMSD also demonstrated a tendency for the higher temperatures to occupy increased RMSD bins (Fig. 2D). This was not seen for RMSF measurements (Fig. 2B, E) where no significant differences were seen as temperature increased, whilst *R*_*G*_ showed a slight increase at higher temperatures later in the simulation, but the difference was < 0.1 A and thus likely insignificant (Fig. 2C, G).

### Increased RMSD was seen most commonly in monomer PROA

**Fig. 3:**
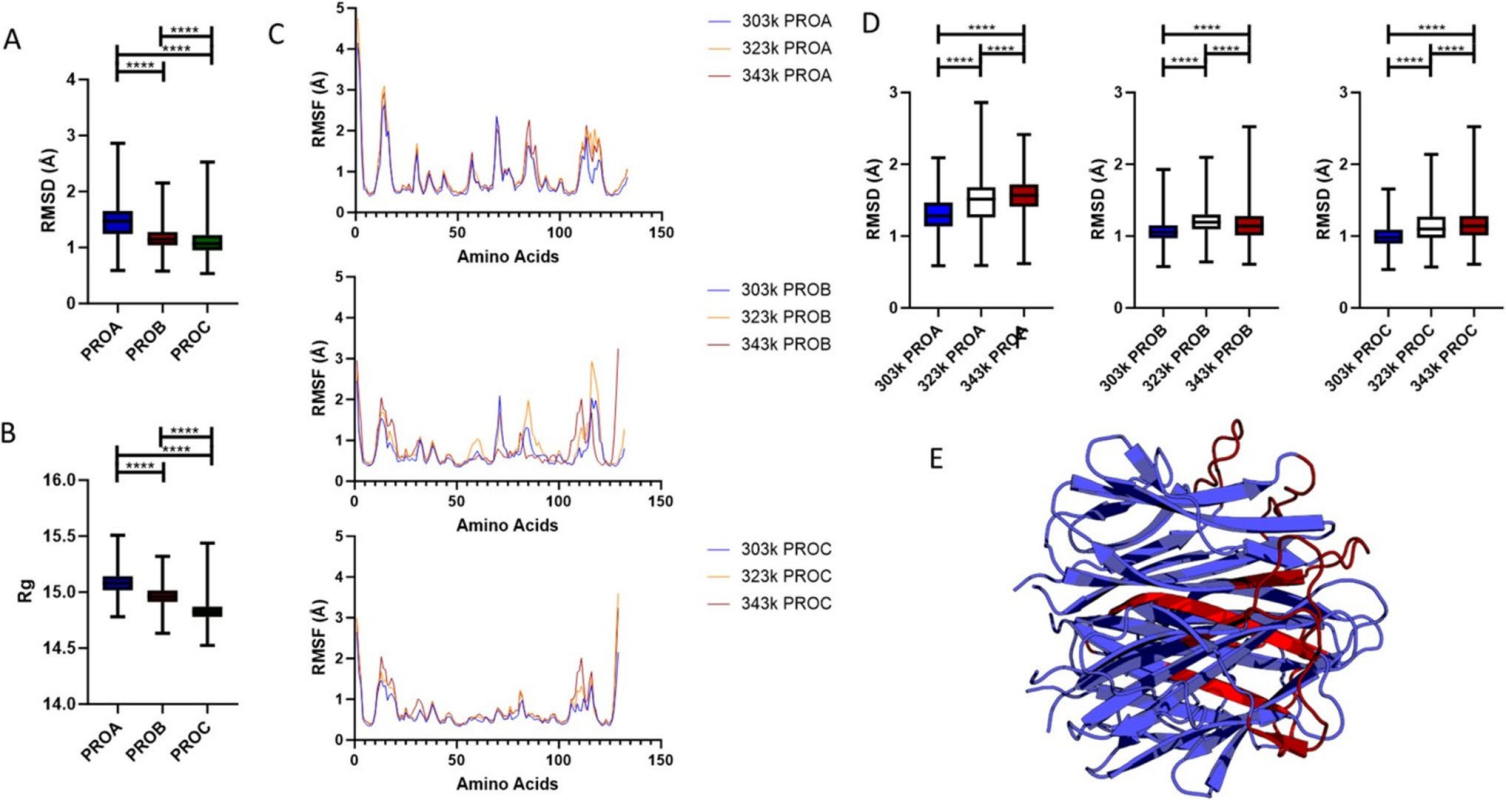
Investigation of monomer eff ects on Trimer show monomer A with a higher RMSD. Panels **A** and **B** show the RMSD and Rg for the monomers across all simulations and temperatures with PROA showing the greatest movement and size, further to this in Panel **C**, increased movement was tracked per aminoacid using RMSF, and increased RMSF was seen at higher temperatures predominantly at 323k. The sites of this increased RMSF were plotted on a crystal model and the sites were all proximal **E, Red** suggesting the end of the molecule is moving together. Finally, the effect of temperature on monomers individually for RMSD was plotted with PROA showing the greatest increase, and PROB the least, while PROC showed consistent increase in RMSD throughout **D**

Monomeric movement was evaluated using RMSD measurements per amino acid per frame and total RMSD calculated by averaging the RMSD values across the trajectory and across the amino acids for each monomer. As can be seen, PROA shows the most significant movement across all simulations at all temperatures (Fig. 3A); the same trend is seen with *R*_*G*_; however, the greatest range of *R*_*G*_ is seen in PROC, suggesting whilst PROA may be the largest monomer, PROC may be the most flexible (Fig. 3B). However, a difference of less than 1 is likely not biologically relevant for *R*_*G*_. Monomeric RMSF values were calculated per amino acid, and little difference was seen in RMSF between amino acids, with the most significant heat-based changes occurring in the regions of AA10-20, AA 80–90, and AA110-120 (Fig. 3C). In contrast, PROB showed higher variability of RMSF, with differences seen throughout the protein most notably at 343 k in the region of AA80-90 and AA105-130, whilst PROC mimicked the patterning seen for PROA with significant changes in movement restricted to specific areas (AA10-20, AA 110–120) (Fig. 3C). RMSD for individual domains at different temperatures showed increased movement, particularly for A and C (Fig. 3D), whilst all increases in temperature facilitated increased movement for each monomer. All the sites highlighted by RMSF analysis cluster on one end of the protein, suggesting this movement may be coordinated between monomers (Fig. 3E).

### Increased temperature alters long range associations between monomers

**Fig. 4:**
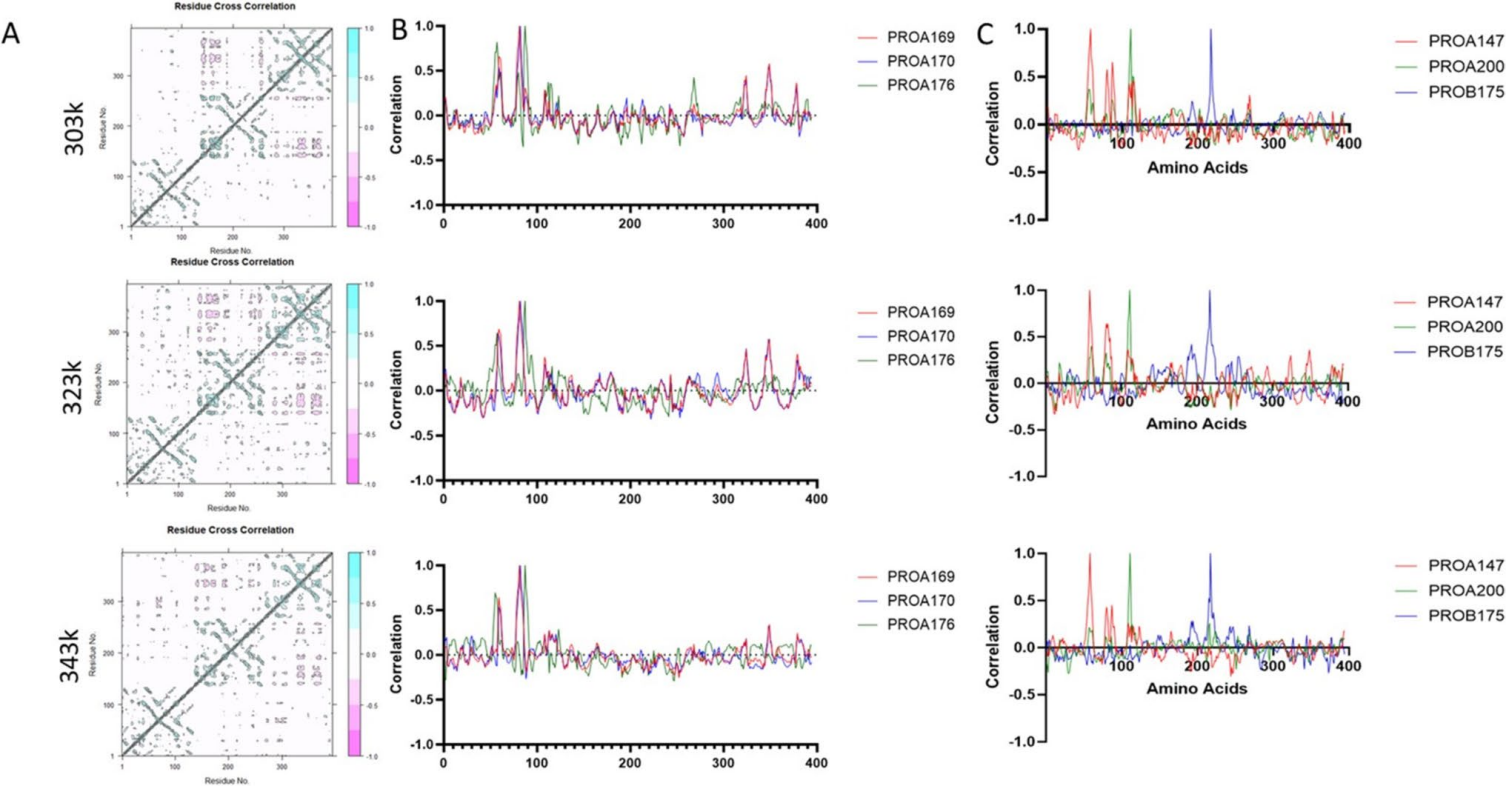
This demonstrates correlative change in the trimer when exposed to increased temperature. The top row represents 303 k, the row below 323 k, and bottom 343 k. The DCCM graph shows at 323 k an increase in negative movement globally which is retained at 343 k. The sites of most significant absolute change were calculated from 303 to 323 k and 343 k and plotted for their long range correlation, and this can be seen in the 2nd column plotting PROA169, PROA170, and PROA176, which show significant correlations in PROA and PROC; however, as temperature increases, these are lessened or lost completely in the case of PROC, suggesting temperature can overcome correlative change. Finally, we examined the correlation changes for the CRP binding amino acids across temperature, highlighting a region at approximately 80 amino acids in sequence, which includes PROA169-PROA176. This suggests the alterations in correlation may have effects on CRP binding

Long range associations were analysed by DCCM (Fig. 4A), PROA169, PROA170, and PROA176 all show strong correlations to the local environment and long range with positive correlations to PROC (AA > 300) (Fig. 4B). As temperature increases this correlation to the long range is lost (343 k, Fig. 4B bottom). Interestingly, these sites correlate strongly with CRP binding sites (PROA 147, PROA200, RPB175) even through increased temperature (Fig. 4C). This suggests they may be part of a correlative node in the protein.

### Structures only found at higher temperatures

**Fig. 5:**
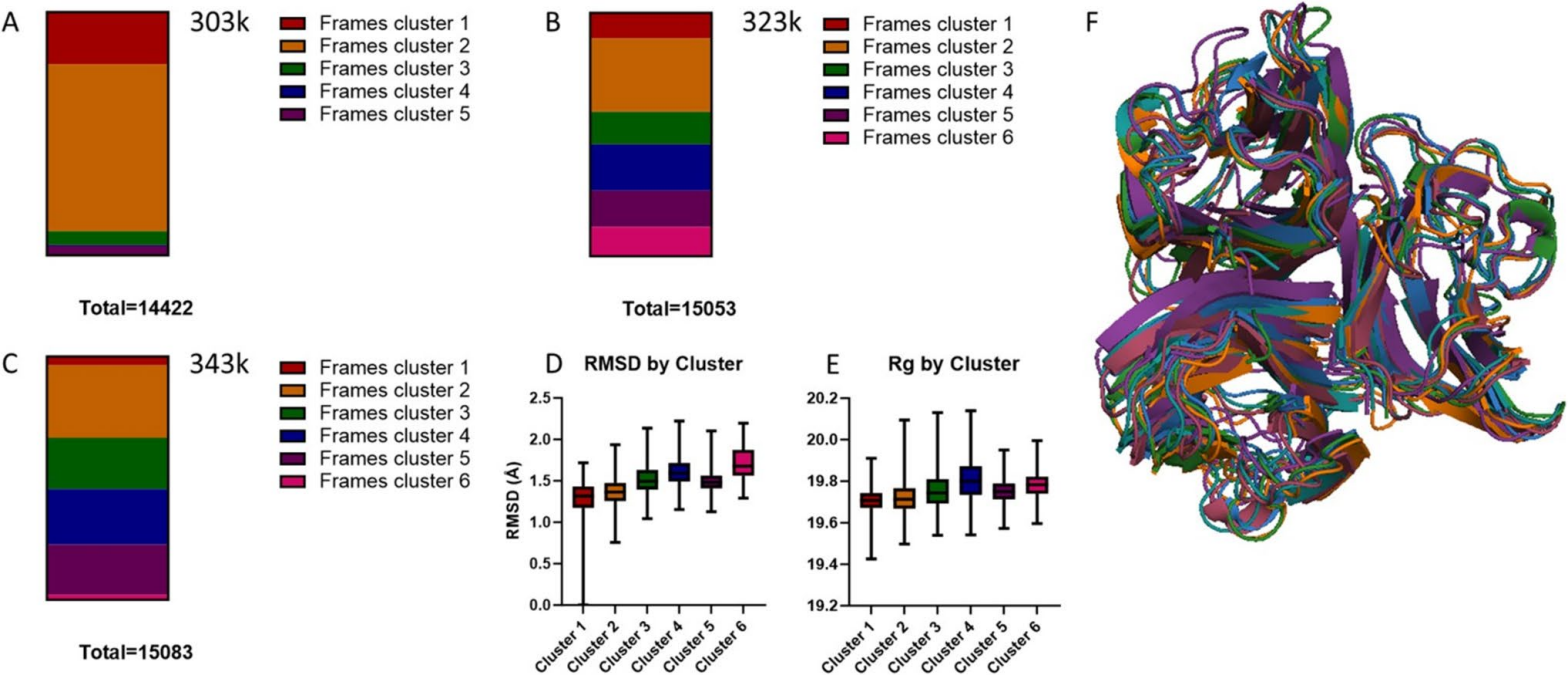
Panel A demonstrates the structures which predominate at 303k, most frames are in clusters 1 and 2 which are shown in panel D to be lowPanel A demonstrates the structures which predominate at 303k, most frames are in clusters 1 and 2 which are shown in panel D to be low RMSD states, whilst at 323k (Panel B) these are less dominant with frames from clusters 3-6 seen. These clusters have higher RMSDs (panel D) and have the greatest Rg (Panel E). At 343k, majority of frames from clusters 3, 4 and 5 are generated, suggesting at the higher temperature structures with higher RMSD and Rgs are found more frequently. Panel F shows an overlay of the clusters aligned by PROA residues 92-120, demonstrating the differences are subtle and appear to be a relaxing and the monomers moving apart.

Analysis by PCA highlighted a number of structures which were more prevalent at higher temperatures; these occupied clusters 3–6 (Fig. 5A-C). These structures were non-identical with clusters 3, 4, and 6 showing higher RMSD values than clusters 1 and 2 (Fig. 5D). In contrast, cluster 5 shows a lower RMSD. Similarly, *R*_*G*_ analysis showed incremental increases from cluster 1 to 3, whilst clusters 5 and 6 show no increase from cluster 4 (Fig. 5E). These clusters, when overlaid, show some significant changes including the loss of alpha helices (lower section, Fig. 5F) in all but clusters 1 and 3, and a general loosening of structure. Interestingly, cluster 4 (shown in purple) shows an abnormal alignment of the loops in the top portion (PROB106-111, PLRRDQ). This suggests there are structures which become more easily accessible in higher temperature or greater energy environments which may be relevant for disease states.

### Structural differences may alter binding kinetics

**Fig. 6:**
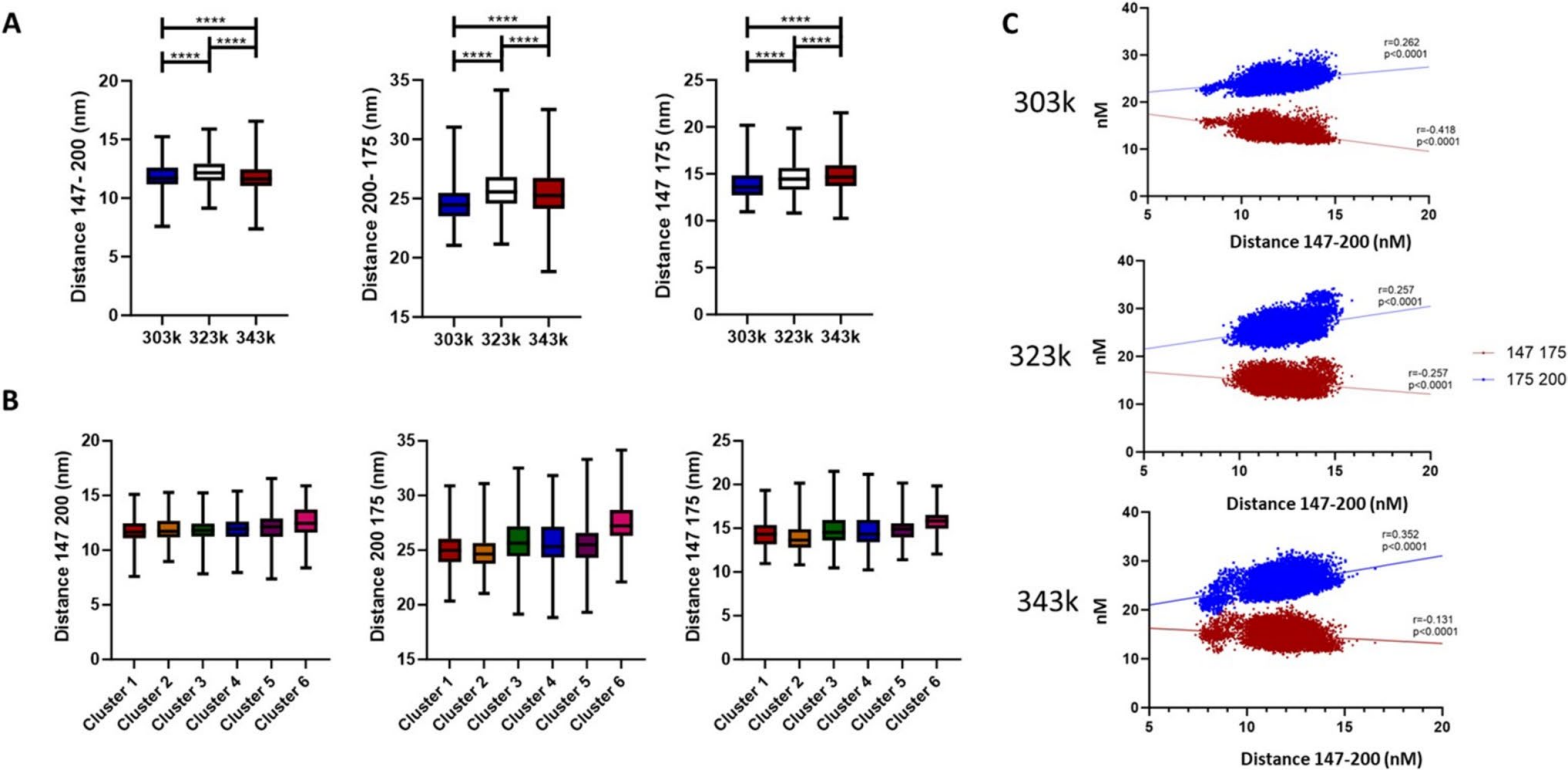
Panel **A** shows the relative distance between the CRP binding amino acids at different temperatures, as can be seen there are significant differences; however, 200–175 and 147–175 appear to be the most affected by increased temperature. Panel **B** shows that these differences do not necessarily effect clustering or reflect the different groupings of structure; however, in the case of 200 175, there is an association with larger distances seen in clusters 3–4, which are more predominant at higher temperatures suggesting potentially this bond is crucially altered in higher energy states. Finally, measuring associations between the distances of 147–200 (the least effected by clustering or temperature) and the other distances showed that as temperature increased, the correlation with 147 175 was lost (R-0.418 to R-0.131), whilst increased temperatures slightly increased the correlation with 175 200 but only at the most extreme temperature 343 k (r 0.262, r 0.352)

To assess the potential for altered binding kinetics specifically within the CRP binding motif, we measured the distance between the amino acids of interest (147, 200, 175) at varying temperatures. Significant differences in the distance between these three amino acids were seen as temperatures increased (Fig. 6A) with the greatest differences seen at 323 k and the greatest change in distance between 200 and 175. Analysing these distances when split for cluster identified the distance between 200 and 175 as that with the greatest variability with cluster 3, 4, and 6 showing increases in average distance, whilst cluster 5 showed an increase in variability. Interestingly, cluster 6 showed increases in all distances, and this was the cluster most associated with the 323 temperature condition and showed a high RMSD but a lower *R*_*G*_ than other high RMSD clusters (Fig. 6B). This suggests that the movement in this cluster may be different leading to different exposures of the molecule. To further investigate the movement of these amino acids, we correlated the distances between all three points. As can be seen, at lower temperatures, a strong negative association is seen with the distance of 147–200 to 147–175 (*R* = − 0.418); however, this is lost as temperature increases (323 k − 0.257, 333 k − 0.131), whilst in contrast, there is a positive association between 147–200 and 175–200 which is unaffected (303 k 0.262, 323 k 0.257, 333 k 0.352). This loss of correlation for 147–200 with 147–175 suggests 147 is significantly affected by these temperature changes (Fig. 6C).

### Potential epitopes cluster on monomer PROA/PROC interface

**Fig. 7:**
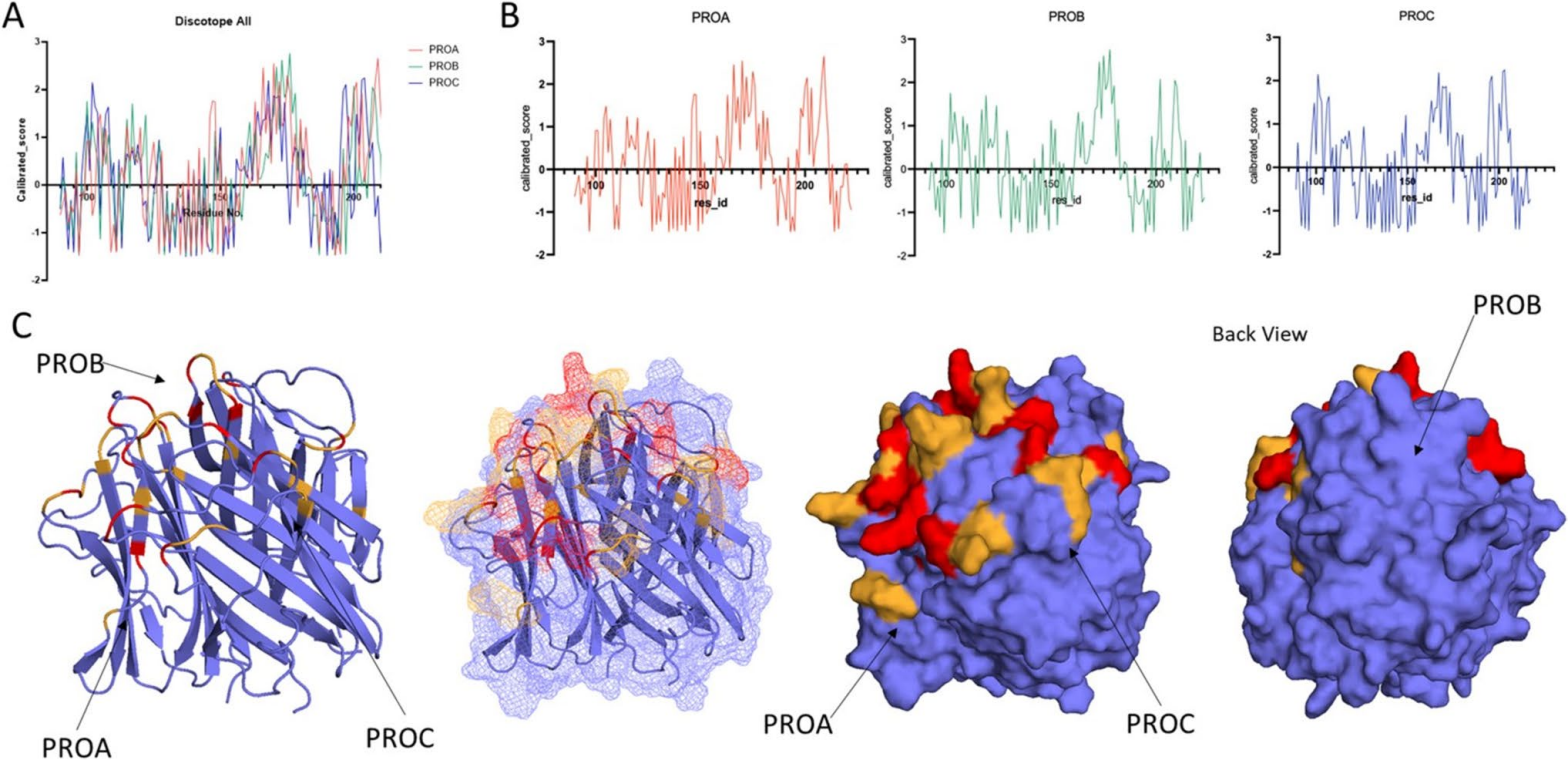
Discotope epitope prediction. Panel **A** demonstrates the calibrated Discotope score of all monomers, the higher the peak, the more likely to be an epitope. The highest peaks are in the region of residues 160–170 and 195–210. Panel **B** demonstrates the calibrated score of each monomer individually (PROA starts at residue 90, PROB 92, and PROC 89); PROA and PROB demonstrate the highest peaks above 2; PROA: 160–180 and 195–210, PROB: 160–180, PROC: 101, 167, and 202–203, suggesting these are likely to be epitopes. Panel **C** illustrates the globular head of C1q trimer with any residue that has a calibrated score of over 2 coloured red and over 1.5 coloured orange. A score over 2 suggests an epitope is most likely, with 1.5 being less likely but still potential. The high potential epitopes from different monomers cluster on the surface increasing the likelihood of them being an epitope

Using Discotope 2.0, we assessed monomers for their potential to house epitopes in the trimeric form. The same motifs were largely seen to be potentially pathogenic with sequences between 150 and 190 housing potential epitopes on PROA and PROB, whilst PROC had generally lower scores (Fig. 7A, B). Interestingly, one site on PROA was positive which was not identified in other monomers (AA 140–150) which contains one of the CRP binding sites, whilst a later peak highlighted a potential epitope surrounding AA PROA200 which also binds CRP. It was shown that these structures cluster together at the sites of transition between monomers (Fig. 7C) primarily on PROA and PROC.

## Discussion

Binding of CRP to C1q through the globular domain has been proven previously [9], whilst localisation of the sites has been shown to be within the C1q A chain [28]. More recently, people have tried to understand the role of C1q’s globular region through a structure-based approach [29]. To our knowledge, no comprehensive simulation of C1q’s globular domain has taken place with the intent to identify the potential for novel structure identification. In our study, we present, to our knowledge, the first atomistic simulation of C1q’s globular domain utilising a heat rap approach to identify rare event structures. We have further identified the potential for variability of CRP’s binding site in these simulations, with alterations in the correlations of the three main binding amino acids PROA145, PROA200, and PROB 175 seen both long range across themselves, with PROA147 showing loss of correlation locally most strongly.

A number of studies have since identified antibodies to C1q which target the globular head domain [30], including a number which block binding of C1q to both IgG and CRP [31]. A recent paper by Duvall et al. [32] shows an scFV with the ability to bind the globular head of C1q abrogates complement activation by apoptotic cells. The intrinsic link between C1q and CRP is perhaps best proven by Sjowall et al. [10] who show solid phase classical complement activation by CRP is abrogated by the addition of CRP-C1q complexes.

Interestingly, monomer A harbours a number of sites vital to the function of C1q, including 2/3 of the CRP binding sites. Its increased movement also likely drives the increased antigenicity seen in the discotope results. The increased RMSD and Rg may also drive some of the structural change associated with altered function, specifically regarding both CRP and DNA binding. Increased movement may either isolate the 2 CRP sites from the 3rd site on PROB, reducing its affinity for the molecule, or expose non-toleragenic sites. Similarly, the movement may alter DNA binding by shielding the DNA binding site on the protein. Correlative relationships with monomer A are also seen in PROB and PROC, which are lost at higher energy states suggesting monomer A may also play a role in holding the trimer together. The increased movement reduces the correlation to the other monomers and could affect function in this manner too.

Using a B cell epitope prediction algorithm, we identified the potential for autoantibodies to the globular head to block CRP binding, by competing for the same sites. This correlates with the data seen by Radanova et al. [31] who identified both the presence of anti-C1q antibodies targeting the globular head and their ability to alter CRP binding in lupus nephritis patients. These antibodies were identified to bind the PROB chain and inhibited both CRP and IgG response of C1q, and it is likely therefore to bind to a site proximal to Tyr175 which has previously been identified to be in both responses. Interestingly, a better signal was seen for binding in these patients when C1q was presented as atrimer rather than monomers which corroborates data from our study showing these antibody binding epitopes clustered at the interface of multiple monomers.

The work identifying novel structures of C1q which may lead to altered function also fits well within lupus literature, who show alterations in C1q function, either through mutation or absence, lead to the development of disease. Point mutations in PROA have been associated with increased levels of lupus [33], whilst further studies have identified C1qA mutations in an African-American pedigree [34] leading to lack of expression and lack of complement activation in these patients. Other studies with C1q alterations [35] identified that mutations in the C1qA gene which alter either expression or functionality led to the onset of juvenile SLE. A further in silico study in 2022 [36] identified a number of C1qA specific missense SNPs, of which 10 were identified to alter structure and function of the protein, several of these mutations are close to sites of interest identified in our study (AA 149, 157, 159), and these may lead to alterations in hydrophobicity and thus alterations in structure.

Whilst raised CRP is not classically seen in SLE, a number of scenarios and roles have been proposed for CRP in SLE [37]. An interesting new paper by Karlsson et al. [38] has shown a new role for CRP in SLE, with the monomeric form binding to extracellular vesicles and acting as an autoantigen. These autoantibodies often associate with nephritis [39], a condition which is also generated by anti-C1q antibodies. Furthermore, levels of these extracellular vesicles are increased in anti-CRP positive patients, and these patients have significantly increased C1q establishing another connection between the two proteins [40]. Other SLE settings also show an increase in CRP, for example, in patients with active serositis [41], where patients have shown higher levels during self-reported flares. High CRP levels are seen in a number of SLE scenarios, for example, in cases of serositis [42] or arthritis; thus, the interaction of CRP and C1q would be a more classical process. It has also been associated with atherosclerotic risk in SLE patients [43].

The normal function of CRP binding to C1q is to modulate complement activity, with papers showing that a dose-dependent interaction in the fluid phase of CRP and C1q takes place and modulates the activation of complement on surfaces [10]. CRP can bind to phosphocholine in an orientation which leaves its C1q binding exposed [44, 45] meaning complement activation can take place as per an antibody-antigen complex; however, it fails to generate the terminal complex perhaps due to the increased interaction with Factor H [10]. Interestingly in SLE, patients frequently present with normal CRP but a low C3 level, and this may be one of the contributing factors, whereas at high CRP levels, this non-canonical classical complement activation is inactivated as the C1q-CRP binding becomes fluid phase and lacks the surface to generate complement activation [10].

It has also been shown recently that heat can directly alter complement activation, with increased or decreasing temperature leading to activation of complement [46, 47] although the role of C1q in either of these is incompletely understood. Patients with lupus suffer from chronic fevers and have local sites of inflammation with significant increases in temperature globally and locally which may lead to structural alterations in protein. This structural change may lead to functional shift, and as such, it is crucial to understand the potential for inflammatory driven structural change and its effect on local functioning.

In conclusion, we present a molecular simulation of C1q measuring its altered binding sites for CRP, showing as increasing energy is applied, there are alterations in structure. This suggests the possibility that these fluctuations may influence the activity of CRP in the context of its C1q binding and may also influence the binding of autoantibodies to C1q.

## Data Availability

All simulation data will be made available under reasonable request to the corresponding author.
